# Evaluating Primitive Reflexes in Early Childhood as a Potential Biomarker for Developmental Disabilities

**DOI:** 10.1111/jpc.70053

**Published:** 2025-04-08

**Authors:** Gerry Leisman, Robert Melillo

**Affiliations:** ^1^ Movement & Cognition Laboratory, Faculty of Social Welfare and Health Sciences University of Haifa Haifa Israel; ^2^ Resonance Therapeutics Laboratory, Department of Neurology University of the Medical Sciences of Havana Havana Cuba

**Keywords:** autism spectrum disorder, developmental disabilities, maturation, paediatric examination, primitive reflexes

## Abstract

We aim to understand better the significance of retained primitive reflexes (RPRs) and examine the effect of RPRs in children, adolescents, and adults, focusing on autism spectrum disorder (ASD) and other neurodevelopmental conditions, as well as examine a basis for future treatment alternatives. We included a history section to better recognise the way that the scientific and medical communities have studied and understood the importance of RPRs. We review findings indicating that aspects of these disorders are related to the presence of functional disconnectivities related to a cortical maturational effect on neuronal networks. Cortical maturational delay within specific networks may lead to enhanced growth and maturation in other networks, resulting in asynchronous development and inconsistency in functional skills. There has been reported an overconnectivity of short‐range, more immature connections and an underconnectivity of long‐range, more mature connectivities. We review the relationship between motor and cognitive impairments and RPRs. A crucial conclusion will be that inhibiting these RPRs is representative of treatment targets.

## 1 Primitive Reflexes: What Are They, and What Is the Controversy?

Primitive reflexes (PR) are neonatal motor and sensory reflexes. Many are present in utero [1] to assist the infant in “birthing itself” [2]. The major role of PRs is to enable the neonate and infant to move and respond to its surroundings before maturation of the motor cortex. The infant needs to feed, move, obtain nourishment, receive protection, and orient to people and objects to stimulate its senses and muscles, thereby generating motor and sensory feedback to activate genes at a foundational level essential for bottom‐up brain development [3]. The regulation of PRs originates from several brainstem areas [2, 3]. The medulla's lower reflexes are thought to be the first to activate, succeeded by reflex regulation linked to the mesencephalon and pons [4].

As higher brainstem regions activate, lower brainstem‐controlled reflexes are suppressed [2, 3], ultimately extending into the brain and neocortex. Numerous investigators have indicated that frontal lobe development facilitates top‐down regulation and suppression of PRs [5]. Degeneration, a frontal lobe lesion, or damage to the corticospinal tract at a more advanced age might result in the re‐emergence of reflexes, termed “frontal release signs” [2, 6], thereby connecting cognition with reflex and motor functions [3, 6]. The debate around retained primitive reflexes (RPRs) pertains not to their existence. PR testing has been a component of standard paediatric neurology assessments for decades. They are a well‐recognised component of assessing effective child development [7, 8]. The controversy pertains to the integration of these reflexes.

In conventional paediatric practice, it is presumed that PRs are largely integrated by the conclusion of the first year [9, 10]. Nonetheless, numerous studies have demonstrated that significant numbers of individuals do not inhibit PRs during the first year of life, with those reflexes present throughout middle childhood and adulthood [2, 3, 11, 12, 13, 14, 15, 16, 17, 18]. It has been shown that neurobehavioral problems or learning difficulties coexist in children, adolescents, and adults with RPRs [2, 3, 16, 17]. Individuals with autism, dyslexia, ADHD, Tourette's syndrome, and other neurobehavioral disorders frequently exhibit RPRs thought to be associated with maturational delays [3, 19]. We assert that if PRs are not integrated within the first year postpartum, they may remain throughout an individual's lifetime, adversely impacting neurobehavioral function. We additionally hypothesise that RPRs are strongly correlated with autism spectrum disorders (ASD) [19, 20] and are significantly linked to motor cortical maturation as well as to movement and cognition [3].

### 1.1 Retained Primitive Reflexes Versus Retuned Primitive Reflexes

It is essential to distinguish between “retained PRs” and “returned PRs” (RtPR). RtPRs have been documented in dementia and Parkinson's disease [21, 22]. RPRs suggest dysfunction of the cortico‐subcortical neural network or potentially a delay in neuronal development. Some investigators have asserted that these reflexes are present in neurotypical populations. The palmomental reflex was observed in 6%–27% of young adults aged 20–50 years [23], and in 28%–60% of individuals over 60 years of age [23]; snouting in 13% of individuals between the ages of 40–57 years [11, 24] and in 22%–33% of those above 60 years [11, 25]; the sucking reflex, linked to “frontal lobe disease,” was found in more than 6% of normal individuals aged 73–93 years [11, 26]. Consequently, there exists contention on the pathogenic importance of these reflexes related to ageing.

### 1.2 The Development of Brain Function in Infancy and Early Childhood Related to Retained Primitive Reflexes

PRs facilitate primary movements, enabling basic engagement with the environment and establishing the foundation for early motor activity [27]. These movements enable newborns to engage with their environment and stimulate receptors and sensory organs. Increased stimulation and sensory feedback is understood to induce gene expression associated with the formation of brain functional connectivities [28]. As neurones increase in size, density, and connectivity, propriospinal projections will ultimately inhibit more primitive lower regions of the brain while stimulating the activation and growth of higher rostral areas of the brainstem and cortex [29]. PRs are ultimately integrated but never eradicated. Ultimately, all reflexes appear to be regulated by the frontal lobe [30, 31].

The existence of these reflexes is a prevalent characteristic among children with ASD and other neurobehavioral problems [20, 32]. In the majority of instances, there exists no discernible damage, injury, lesion, or degeneration. We have considered and discovered support for RPRs signifying a maturational delay in brain regions that normally suppress PRs, particularly reflexes interacting with the frontal lobes [3].

However, frontal lobe maturational delays can be reflected by the RPRs and additional delays in postural reflex development, which could lead to delays of various sensory‐motor milestones like crawling and walking [33]. Maturational delay can also be related to the lack of development of executive functions, a hallmark of neurodevelopmental disorders [34, 35]. The reduction, lack, or absence of environmental factors that typically foster development, growth, and neuroplasticity in higher brain regions generally results in the lack of integration of PRs and the manifestation of postural reflexes [33]. RPRs may indicate a developmental delay [33], and asymmetric persistence will signify a maturational delay in the developing brain and may imply, contingent upon the timing, aberrant asymmetrical cortical hemispheric development [36, 37].

### 1.3 Bottom‐Up Versus Top‐Down Regulation and Retained Primitive Reflexes

Since the motor cortex is still immature and underdeveloped, there is no volitional motor control present in the neonate. The infant needs to be able to move to survive, and motor activity activates the senses, sending feedback to the brainstem, in turn promoting neuroplasticity [38]. This promotes growth in higher levels of the brainstem, such as the pons, which, through proprioceptive connections, will descend into the medulla inhibiting PRs from that level [38, 39]. This will also release more sophisticated PRs, allowing for more complex interaction with the environment and more activation of senses, which will provide feedback and promote neuroplasticity at higher levels, such as the mesencephalon [38]. This will progress through the brainstem and ultimately into the neocortex, where sensory feedback promotes growth of sensory areas and ultimately the temporal–parietal–occipital cortex [38], a major association area promoting sensory integration [40].

Sensory and motor activity feedback will also promote the growth of the frontal and prefrontal cortex [41], growing out of the motor cortex. The neocortex, especially the prefrontal cortex, projects fibres to the brainstem to achieve “top‐down” control of the nervous system and its bodily functions [42]. It is also thought that the frontal lobe, through top‐down integration, inhibits the PRs [19]. Later in life, if there is damage, injury, or degeneration of the frontal lobes, one can observe “frontal release signs” exhibited as the return of PRs such as the Babinski reflex [43]. We think that if the PRs are not inhibited, they will continue to promote bottom‐up interference, prevent maturational completion, and ultimately prevent appropriate top‐down regulation [2, 3]. This can lead to RPRs and a global dysregulation of nervous, immune, endocrine, and autonomic systems [44].

## 2 The Effect of Retained Primitive Reflexes on Motor and Cognitive Development in ASD?

ASD is presently defined by deficits in social interaction, communication, and behavioural flexibility. ASD is characterised as a neurodevelopmental disorder marked by impairments in language, executive function, social interaction, and emotional regulation [45]. While there is agreement on the disorders constituting ASD, including autistic disorder, Asperger's disorder, and Pervasive Developmental Disorder‐Not Otherwise Specified (PDD‐NOS) as the most prevalent examples, as well as the associations with Rett's syndrome and disintegrative disorder, significant controversies persist concerning the definitions of ASD and the delineation from other manifestations of these disorders. We are increasingly acknowledging the impact of motor skills on other developmental domains, including language and social cognition.

RPRs can hinder normal growth and are associated with impediments in children's educational and social experiences. Psychomotor development can also be affected. Mature responses in psychomotor development in childhood can only occur once the central nervous system (CNS) has reached an appropriate level of maturity. The process entails a change from reflexive brainstem responses to cortically‐based responses. PRs are highly associated with both social (e.g., communicative gestures) and object interaction (i.e., actions) in infancy. This indicates that reduced scores in the primitive reflex evaluation, signifying increased reflex persistence, correlate with lower motor repertoire scores, irrespective of the infant's age. Postural reflexes are sophisticated response mechanisms that govern balance, coordination, and sensory‐motor maturation. RPRs are correlated with developmental delays related to ADHD, autism, learning disabilities, and sensory processing disorders.

Children with autism frequently struggle with executing skillful movements and exhibit a limited repertoire of gestures [46]. Motor deficits in early childhood have been proposed as predictors of the fundamental impairments associated with ASD. Motor function development is associated with automated processes whereby newborns progressively mature by suppressing more rudimentary motor behaviours. These findings imply that motor deficits in early childhood are predictive of the primary impairments associated with ASD. This concept has been examined in infant siblings of children diagnosed with ASD, who possess an elevated probability of developing the disorder. Research on motor development in high‐risk infants aged between 3 and 6 months indicated that 67%–73% of autistic infants with early motor deficits later displayed communicative problems. Progress in motor development is associated with an automated process whereby the newborn eventually matures by suppressing more basic motor patterns [47].

Movement disturbances in infancy were considered by Teitlebaum and colleagues [48] to be “reflexes gone astray” serving as potential early indications of ASD. Their study indicated that some children exhibited RPRs beyond infancy, whereas others displayed the emergence of some RPRs significantly later than expected throughout infancy. They noted that the asymmetric tonic neck reflex may persist in individuals with ASD. Another reflex, head verticalization in response to body tilt, was found to be absent in a subgroup of infants identified as “autistic‐to‐be.” They proposed that these reflexes may serve as ASD indicators and might be utilised by paediatricians to assess neurological impairment. Teitelbaum and associates [49] also demonstrated that infants who would later develop ASD exhibited a distinct set of movement pattern differences as early as 4–6 months of age.

Among the postnatal PRs that decrease as development progresses are the Galant and Moro reflexes. Konicova and Bob [50] examined school‐aged children with ADHD (ages 8–11) presenting with Galant and Moro reflexes evaluated against a control group of the same age. Children with ADHD manifested a markedly greater prevalence of Moro and Galant responses in comparison to the control group.

Callcott [51] indicated that learning and cognitive difficulties in childhood are related to movement skills, including early academic preparedness. Callcott examined the prevalence of the Asymmetrical Tonic Neck Reflex (ATNR) and assessed the motor skills of pre‐primary school Western Australian Indigenous children. She reported that 65% of the Indigenous children studied exhibited moderate to high levels of ATNR, which correlated with academic achievement.

The United Kingdom's Millennium Cohort Study [52] found a correlation between delayed motor milestones at 9 months and significantly diminished cognitive function in five‐year‐olds. The Australian Early Development Index [53] revealed that approximately 25% of school‐aged children are at ‘risk’ in their cognitive and physical development. A relationship was found by Williams and Holley [54] between cognition and motor development. They investigated how infant motor experiences in early life influence cognitive abilities essential for academic achievement. The standard progression of motor skills and gestures requires effective inhibition of PRs, particularly those associated with the mouth and hand.

Chinello and colleagues [20] explored the correlation between RPRs, motor repertoire, and autistic‐like characteristics in parents of infants aged 12–17 months. Regardless of age, RPRs influenced the motor skills of infants and evidenced a strong correlation with autistic behaviours of the parents. A study conducted in Egypt [55] examined 206 students aged 9–10 years for dyslexia. The study employed comprehensive history‐taking and neurological examinations, focusing on cerebellar indications, PRs, dyslexia, and apraxia assessments. Findings revealed that 22 students (10.67%) were identified as dyslexic. Dysdiadochokinesia, asymmetric tonic neck reflex, neck retraction, palmar reflexes, and apraxia were markedly elevated in individuals with dyslexia. The reversal of letters, the persistence of the ATNR, apraxia, and neck retraction were found to be strong predictors of dyslexia.

Numerous investigators have observed a correlation between incoordination and clumsiness, particularly in posture and gait, and ADD/ADHD, ASD, and other neurobehavioral disorders [56, 57]. Gait and motor disturbances were principally compared to those cerebellum‐related [58]. The most frequent childhood neurobehavioral disorder comorbidities are developmental coordination disorder (DCD), “clumsiness,” or motor incoordination [59]. Incoordination typically pertains to the muscles responsible for posture, locomotion, or gross motor functions. Not infrequently, fine motor skills are also compromised [60]. Wisdom and associates [61] noted both distinctions and commonalities between ASD and DCD, indicating that the DCD cohort had superior emotion detection, theory of mind, and gross and fine motor coordination compared to the ASD cohort while demonstrating similar response inhibition capabilities. Wisdom and colleagues [61] observed that when categorised by symptom intensity, children with ASD deemed “more able” exhibited no significant differences in any measures compared to children with DCD, in contrast to those classified as “less able.”

## 3 Discussion

PRs provide a developmental function, enabling neonates to counteract gravity and facilitate voluntary movement through the integration of motor activity in the early months of life. Mature responses in a child's psychomotor development can only manifest when maturity has been obtained in the CNS. The process involves shifting from brainstem‐based reflexes to cortically regulated responsivity. With impairment in the process of the development of integrated movement, the child may exhibit immature motor skills, evidenced by difficulties in maintaining balance, running, and cycling, oftentimes resulting in clumsiness. The child may experience difficulties with throwing and catching. Psychomotor abnormalities, sometimes referred to as minimal brain dysfunction can alter and impede a child's natural developmental trajectory. Initial indicators may manifest in early childhood; however, several signs become apparent later, such as learning and behavioural challenges during the preschool years. RPRs and the cognitive or behavioural challenges encountered by children upon reaching school age are correlated.

As a child develops, these PRs are supplanted by more advanced reflexes. The PRs that were once essential become inhibitory or redundant. The grasping reflex enables an infant's small hands to grasp objects as required; nevertheless, as the hands and fingers expand, this reflex must diminish for the proper development of fine motor abilities. Children who maintain the clutching reflex throughout toddlerhood rather than progressing to the “precision grip” may encounter difficulties in holding a crayon, turning a page, or self‐feeding at the appropriate developmental stage.

RPRs may be maintained for several reasons. Studies have found associations between traumatic delivery [62], adverse neonatal conditions [62], and recurrent ear infections in early childhood [46] with potential changes in a child's developmental trajectory. Infants that bypass developmental milestones, such as walking before crawling, may be more prone to RPRs, similar to infants not receiving sufficient “tummy time.” Consequently, PRs can serve as an early and effective instrument for evaluating the integrity of the CNS of full‐term neonates. Later in the first year of life, as the CNS matures, these responses become increasingly difficult to elicit, coinciding with the emergence of voluntary motor activity. RPRs are frequently observed in children with ADHD, autism, and cerebral palsy and may serve as early indicators of developmental delays or nervous system dysfunction. PRs are critical in neonatal and infant neurological assessment. A sucking reflex that is absent or impaired serves as an indirect sign of neonatal neurological maturity. An aberrant sucking reflex, when accompanied by further symptoms of CNS involvement, indicates a potential malfunction of the basal ganglia or brainstem.

## 4 Conclusion

RPRs throughout early development and adulthood are linked to brain injury and neurobehavioral disorders. Their existence has also been noted in many childhood functional neurological disorders without identifiable neurological injury or pathology. We think that the manifestation of RPRs indicates the existence of a maturational delay, also evidenced by structural and functional nervous system, behavioural, and cognitive differences when compared to otherwise neurotypical children.

The preservation of PRs may serve as an early indicator of ASD, which, in conjunction with initial attentional indicators, could aid in delineating the developmental trajectory of the larger autism phenotype in infancy. This approach posits that minor deviations in the initial phases of development, such as with RPRs, may have detrimental cascading effects on subsequent motor function as well as various other functions, including social and communicative behaviour and object exploration.

With RPRs persisting longer than the typical developmental timeframe, they may impede maturation and diminish the brain's capacity to interpret sensory information efficiently. The continuation of PRs beyond the typical duration of 12 months disrupts further development.

In conclusion, we propose that ASDs are partially linked to delays in maturation rather than structural pathology or damage. We think that the existence of RPRs and the potential delays in or absence of developmental milestones may serve as the earliest indicators of developmental delays in children, especially those with ASD. Therefore, given the relative simplicity of examining basic reflexes by both parents and paediatricians, we propose that the persistence of these reflexes could serve as a potentially valuable indicator for advancing more effective early identification and screening methods.

## Ethics Statement

The authors have nothing to report.

## Consent

The authors have nothing to report.

## Conflicts of Interest

The authors declare no conflicts of interest.

## Data Availability

The authors have nothing to report.
